# Barriers and facilitators to implementing a pragmatic trial to improve advance care planning in the nursing home setting

**DOI:** 10.1186/s12913-019-4309-5

**Published:** 2019-07-29

**Authors:** Jennifer A. Palmer, Victoria A. Parker, Vincent Mor, Angelo E. Volandes, Lacey R. Barre, Emmanuelle Belanger, Phoebe Carter, Lacey Loomer, Ellen McCreedy, Susan L. Mitchell

**Affiliations:** 1 Harvard Medical School, 25 Shattuck Street, Boston, MA 02215 USA; 2 Hebrew SeniorLife, Hinda & Arthur Marcus Institute for Aging Research, 1200 Centre Street, Roslindale, MA 02131 USA; 3 Beth Israel Deaconess Medical Center, Department of Medicine, East Campus, Yamins 419, 330 Brookline Avenue, Boston, MA 02215 USA; 4 Peter T. Paul College of Business and Economics, University of New Hampshire, 10 Garrison Avenue, Durham, NH 03824 USA; 5 Center for Gerontology and Healthcare Research, School of Public Health, Brown University, 121 S Main Street, Providence, RI 02903 USA; 6 Department of Health Services, Policy, and Practice, School of Public Health, Brown University, 121 S Main Street, Providence, RI 02903 USA; 7 Providence Veterans Administration Medical Center, Center of Innovation in Health Services Research and Development Service, 830 Chalkstone Avenue, Providence, RI 02908 USA; 8 Massachusetts General Hospital, Section of General Medicine, 55 Fruit Street Gray 7-730, Boston, MA 02114 USA

**Keywords:** Pragmatic trial, Implementation, Nursing homes

## Abstract

**Background:**

The PRagmatic trial Of Video Education in Nursing homes (PROVEN) aims to test the effectiveness of an advance care planning (ACP) video intervention. Relatively little is known about the challenges associated with implementing ACP interventions in the nursing home (NH) setting, especially within a pragmatic trial. To address this research gap, this report sought to identify facilitators of and barriers to implementing PROVEN from the perspective of the Champions charged with introducing the ACP video program delivery to patients and families.

**Methods:**

In semi-structured telephone interviews at 4 and 15 months of the 18-month implementation period, ACP Champions at all PROVEN intervention facilities (*N* = 119) were asked about their perceptions of program implementation. Forty interviews were purposively sampled, transcribed, and analyzed using a hybrid deductive/inductive approach to thematic analysis incorporating the Consolidated Framework for Implementation Research’s domains: Intervention Characteristics (IC), Inner Setting (IS), Characteristics of Individuals (CI), Outer Setting (OS), and Process (P).

**Results:**

Implementation facilitators identified by Champions included: the intervention’s adaptable mode of presentation and minimal time burden (IC) as well as the program’s customizable delivery to patients and families and opportunity for group reflection on implementation among ACP Champions (P). Barriers included mandated protocol-driven aspects of the program (OS), limited time to deliver the intervention (IS), and lack of perceived relevance and emotional readiness for ACP amongst stakeholders (CI).

**Conclusions:**

Despite the promise of PROVEN’s intervention for improving ACP in nursing homes, unchangeable setting and characteristics of Champions, patients, and family members presented implementation barriers. Researchers need to engage all program participants (i.e., facility staff, patients, and families), in addition to corporate-level stakeholders, in early pragmatic trial design to minimize such obstacles. Further, despite the facilitating nature of PROVEN’s implementation processes, the study encountered tension between scientific rigor and real-world demands. Researchers need to optimize the real-world authenticity of pragmatic trial design while avoiding excessive implementation protocol deviations.

**Trial registration:**

ClinicalTrials.gov Identifier: NCT02612688. Registered 19 November 2015.

**Electronic supplementary material:**

The online version of this article (10.1186/s12913-019-4309-5) contains supplementary material, which is available to authorized users.

## Background

Nursing home (NH) patients often receive aggressive and costly treatments that may be of limited clinical benefit and inconsistent with their preferences [1–4]. Advance care planning (ACP) within this setting has the potential to promote more preference-based, higher quality, and cost-effective care [5].

While important to rigorously test the impact of ACP programs, NHs are challenging environments in which to conduct traditional randomized clinical trials (RCTs) which require homogeneous and carefully controlled study conditions to establish efficacy. Indeed, there are few well-powered traditional RCTs testing ACP interventions in this setting [6–9]. Traditional RCTs are also limited in their generalizability to real-world settings [10]. Pragmatic clinical trials (PCTs) conducted in partnership with health care systems are designed to test interventions as they would be done under real-world circumstances. A number of relatively small PCTs have been completed in the NH setting, but none of these have specifically focused on ACP interventions [11–21].

Intervention implementation can be a particular challenge in a PCT, as protocol compliance relies on usual care providers rather than a research team. Limited intervention uptake by these providers - constrained by intensive, competing demands of routine care - can translate into “implementation error”. Such error (i.e., an invalidating degree of deviation from an implementation protocol) may erroneously appear to reflect ineffectiveness [22].

Little is known, however, about the factors influencing intervention implementation (i.e., facilitators and barriers) in NH-based RCTs related to ACP interventions or in similar types of PCTs. Notable exceptions are two qualitative studies examining facilitators of and barriers to two multicomponent interventions: 1) the INTERACT program (already tested through an RCT) [23] and 2) the “COSMOS” (i.e., COmmunication, Systematic assessment and treatment of pain) trial (currently being tested within a PCT) [24]. These qualitative studies point to several similar implementation barriers including significant demands for organizational change required by the interventions [25, 26].

The Consolidated Framework for Implementation Research (CFIR) offers a useful framework for developing this literature further. CFIR is a conceptual framework composed of a well-established set of implementation constructs [27]. It is strongly grounded in the implementation science literature [28] and is useful in comparing implementation findings across varying interventions. Accordingly, this report uses CFIR in its analysis of the implementation experience of the PRagmatic trial Of Video Education in Nursing homes (PROVEN). PROVEN is the first PCT to be conducted in partnership with NH health care systems (HCS) (*N* = 360 NHs) to test the effectiveness of an ACP intervention (specifically, a video education program). Guided by CFIR constructs, we leveraged qualitative interview data with PROVEN Champions charged with delivering the ACP intervention to answer the following research question: “What are the barrier and facilitators Champions face in implementing a pragmatic trial to improve advance care planning in the nursing home setting?”

## Methods

### PROVEN trial overview

Brown University’s Institutional Review Board approved the conduct of the PROVEN trial and determined that the nursing home providers were not engaged in human subjects research.

As mentioned, PCTs strive to assess intervention effectiveness as implemented in real-world conditions. To emulate a pragmatic design, PROVEN’s implementation was managed primarily by the HCS leaders, similar to any new clinical program roll-out in their system. As such, ACP champions, staff, patients, and families were unaware the ACP video program was part of a research trial. Additional pragmatic features included: 1) permitting all patients in intervention facilities to be eligible for the program, 2) allowing intervention facilities to customize implementation as local needs dictated, and 3) capitalizing on already collected and electronically integrated clinical data to assess facility-level implementation [29].

PROVEN was conducted in partnership with two for-profit U.S. HCS which, at the start of the study, together operated a total of 456 facilities in 32 states. Eligible facilities needed to have a bed size larger than 50, to provide care to both short and long stay patients, and to be free of organizational or regulatory compliance difficulties (as determined by corporate leaders). Facility randomization consisted of a two-phase stratification process: 1) stratification by HCS given differing corporate-level characteristics and 2) stratification into terciles related to the trial’s primary outcome (hospitalizations/person-day alive for patients with advanced dementia or pulmonary disease). The resulting distribution of intervention versus control facilities in total and across HCS was: *N* = 119 intervention/*N* = 241 control; HCS1: *N* = 98 intervention/*N* = 199 control; HCS2: *N* = 21 intervention/*N* = 42 control. PROVEN commenced in March 2016 and was rolled out in three implementation waves through May 2018.

Briefly, the intervention consisted of a suite of five videos (~ 6–10 min) that presented a similar framework for choosing preferences and decisions for health care, i.e., preferences and decisions amongst intensive medical care, basic medical care, or comfort care. Each video customized this framework to the particular situation of patients/families: 1) General Goals of Care (providing descriptions of each type of care with narration and visual images), 2) Goals of Care for Advanced Dementia (providing similar information with family members as the target audience), 3) Hospice (providing detail on hospice care options at the end of life), 4) Hospitalization (providing detail on hospital care options), and 5) Advance Care Planning for Healthy Patients (providing basic education on advance care planning for patients with time-limited treatment needs).

At each intervention facility, one or two “ACP Champions” (most often social workers) were assigned and trained to offer a video to all patients or their family members within 7 days of admission (short-stay patients), every 6 months (long-stay patients (length of stay > 100 days)), and upon readmission (short-stay and long-stay patients) over an 18-month period. Videos could be viewed by patients or families on a tablet device or on-line. Champions were instructed to document the introduction of the intervention in a special report form embedded in the electronic medical record (EMR) each time they offered a video, and if offered, whether or not the video was shown.

Training of the ACP Champions was designed and conducted cooperatively by the research team and NH HCS leadership and occurred at a centralized in-person half-day event for the smaller HCS (HCS2) and by webinar for the larger HCS (HCS1). In addition to training Champions strictly in the ACP Video program protocol, trainers also instructed Champions on how to use the ACP videos as a supplement to rather than a replacement of usual advance care planning discussions.

Throughout the implementation, HCS leadership provided Champions with monthly feedback reports generated by the research team that included quantitative measures of adherence (i.e., proportion of patients/families offered a video) based on the EMR report data. These leaders, along with research team members, also conducted regular group conference calls attended by Champions from multiple facilities to share their experiences and problem-solve mutual implementation barriers.

Control facilities conducted their usual ACP practices, and were unaware they were part of a research study. The primary trial outcome was hospital transfer rates per person-day over 12 months, as ascertained from Medicare Claims data obtained directly from Centers for Medicare and Medicaid Services.

### Setting & participants

Semi-structured telephone interviews at 4 and 15 months into the 18-month intervention implementation period with all facility Champions (*N* = 119 facilities) served to better understand perceived facilitators and barriers to program implementation. Whenever possible, the same Champion was interviewed at both time periods. These qualitative interviews and subsequent analyses were auxiliary to PROVEN’s main research aims assessing the intervention’s effectiveness. Among the 119 intervention facilities, interviews were purposively sampled from facilities that were in the top and bottom terciles (N ≈ 40 per tercile) for new admission rates in 2015 based on data available to the research team. This approach was intended to optimize balance of short-stay patients versus long-stay patients among sampled facilities.

### Data collection / measures

Both 4-month and 15-month open-ended interview guides were developed inductively for this study (Additional file 1 and Additional file 2) and included questions addressing the following implementation domains: 1) Champions’ perspective of the training experience and preparedness to implement the program, 2) ACP practices at the facility prior to the program, and 3) Champions’ perspectives of the implementation experience (e.g., successes, challenges, and reactions from patients, families, and non-champion staff). The 15-month interview included additional questions (e.g., about suggested program improvements and whether Champions would recommend the program to other facilities).

### Conceptual framework

The Consolidated Framework for Implementation Research (CFIR) guided the deductive analysis of the Champion interviews [30]. CFIR’s conceptual domains are: 1) Intervention Characteristics (i.e., characteristics of the ACP video program), 2) Inner Setting (i.e., NH), 3) Characteristics of Individuals (i.e., Champions, patients, and families characteristics), 4) Outer Setting (i.e., mandated program requirements), and 5) Process (i.e., implementation efforts). Table 1 presents CFIR’s domains and their nested constructs found relevant to PROVEN. For each construct, published CFIR definitions are presented [30] as well as how we operationalized them for PROVEN.

**Table 1:** Operational Definitions by Consolidated Framework for Implementation Research (CFIR) Constructs

CFIR Construct Definition^a^	Operational Definition
DOMAIN 1: Intervention Characteristics	
Evidence Strength & Quality	
● Stakeholders’ perceptions of the quality and validity of evidence supporting the belief that the intervention will have desired outcomes.	● Perceived quality, effectiveness, and validity of the ACP videos in facilitating ACP conversations.
Relative Advantage	
● Stakeholders’ perception of the advantage of implementing the intervention versus an alternative solution.	● Perception that the ACP videos were more effective than other ACP methods.
Adaptability	
● The degree to which an intervention can be adapted, tailored, refined, or reinvented to meet local needs.	● Perceived extent to which ACP video (e.g., modes of presentation) can be customized to individual patient needs.
Cost	
● Costs of the intervention and costs associated with implementing the intervention including investment, supply, and opportunity costs.	● Perception that ACP videos consume facility resources.
DOMAIN 2: Inner Setting	
Available Resources (within Readiness for Implementation)	
● The level of resources dedicated for implementation and on-going operations, including money, training, education, physical space, and time.	● Perceived availability of organizational resources for ACP video program implementation.
Networks & Communications	
● The nature and quality of webs of social networks and the nature and quality of formal and informal communications within an organization.	● Opportunities for communication between Champions and other facility staff about the ACP program.
Compatibility (within Implementation Climate)	
● The degree of tangible fit between meaning and values attached to the intervention by involved individuals, how those align with individuals’ own norms, values, and perceived risks and needs, and how the intervention fits with existing workflows and systems.	● Impact of how well the ACP video program could be integrated into established workflow upon implementation.
DOMAIN 3: Characteristics of Individuals	
Knowledge & Beliefs about the Intervention	
● Individuals’ attitudes toward and value placed on the intervention as well as familiarity with facts, truths, and principles related to the intervention.	● Champions’ and patients’/family members’ personal attitudes towards and familiarity with ACP.
Individual Stage of Change	
● Characterization of the phase an individual is in, as s/he progresses toward skilled, enthusiastic, and sustained use of the intervention.	● Perceptions of patient/family level of emotional readiness to participate in the ACP video program.
DOMAIN 4: Outer Setting	
External Policy & Incentives	
● A broad construct that includes external strategies to spread interventions, including policy and regulations (governmental or other central entity), external mandates, recommendations and guidelines, pay-for-performance, collaboratives, and public or benchmark reporting.	● Perceived influence of mandates regarding ACP video program implementation relayed by corporate leaders (but actually driven by trial design).
DOMAIN 5: Process	
Engaging	
● Attracting and involving appropriate individuals in the implementation and use of the intervention through a combined strategy of social marketing, education, role modeling, training, and other similar activities.	● Perceived effectiveness of Champion training and opportunities to engage other facility staff members in the program.
Executing	
● Carrying out or accomplishing the implementation according to plan.	● Ways in which Champions adhered to or customized the implementation process as originally planned.
Reflecting & Evaluating	
● Quantitative and qualitative feedback about the progress and quality of implementation accompanied with regular personal and team debriefing about progress and experience.	● Perceptions of ongoing feedback on program implementation provided by HCS leadership (e.g., through cross-facility conference calls).

^a^CFIR Construct definitions are cited verbatim from: https://cfirguide.org/constructs/

*ACP* advance care planning, *HCS* health care system

#### Domain 1: Intervention Characteristics

There are four relevant constructs within the CFIR Intervention Characteristics domain: *evidence strength and quality*, *relative advantage*, *adaptability*, and *cost*. *Evidence strength & quality* refers to the perceived level of evidence supporting the intervention’s effectiveness as derived from a number of sources (e.g., literature, guidelines, or stakeholders’ experiences) [30]. We operationalized this construct as the Champions’ perception of the quality, effectiveness, and validity of PROVEN’s videos in facilitating ACP conversations. CFIR defines *relative advantage* as the perceived benefit of an intervention over an alternate approach to the same problem. We operationalized this construct as the Champion’s perception about whether the PROVEN video program was more effective at improving ACP compared to other approaches. The construct of *adaptability* pertains to how much an intervention can be refashioned to address local needs. Within PROVEN, we conceptualized *adaptability* as how much Champions felt the ACP video program could be customized to their needs and those of patients/families. Finally, the *cost* construct considers resource investment and opportunity costs associated with an intervention. Within the PROVEN context, it referred to the perceived degree to which the intervention consumed facility resources, including the time and effort of the Champions.

#### Domain 2: Inner Setting

Within the Inner Setting domain, the constructs of *available resources, networks and communications*, and *compatibility* were considered. One construct nested within Inner Setting is *readiness for implementation,* in which *available resources* (i.e., organizational dedication of resources to intervention operations) is a sub-construct. We operationalized this sub-construct as the Champions’ perceptions of the availability of organizational resources (e.g., staff time and effort) for ACP video program implementation. The *networks & communications* construct relates to the nature and quality of the organization’s communication systems. In PROVEN, it related to the communication amongst Champions and other staff. CFIR’s construct of *implementation climate* has a sub-construct labeled *compatibility* that refers to alignment between an intervention and organizational climate and systems. We operationalized *compatibility* as how well Champions felt the ACP video program could be integrated into the facility’s established workflow.

#### Domain 3: Characteristics of Individuals

CFIR interprets “individuals” as those involved in implementing the program (i.e., the facility Champions). However, our inductive analysis revealed that the concept of “individuals” should be extended to the end-users (i.e., patients/family members). The *knowledge and beliefs about the intervention* construct in this domain pertains to cognition, that is, individuals’ attitudes towards and familiarity with an intervention. In PROVEN, this construct pertained to Champions’ attitudes towards and familiarity with ACP and those they perceived in patients and family members. *Individual stage of change* is a CFIR construct that captures emotional features, that is, the readiness of an individual (e.g., in a skilled or enthusiastic manner) to use an intervention. In PROVEN, this was interpreted as the patients’ and family members’ emotional readiness (as perceived by the Champion) to participate in the ACP video program.

#### Domain 4: Outer Setting

Within the Outer Setting domain, CFIR defines the construct of *external policies and incentives* as relating to strategies used by centralized bodies to disseminate the intervention (e.g. external mandates). In PROVEN, this was operationalized as the Champions’ perception of how the mandate passed down to them by HCS leaders in how to offer the ACP program influenced its implementation.

#### Domain 5: Process

CFIR’s constructs in the Process domain included: *engaging*, *executing*, and *reflecting and evaluating*. CFIR’s *engaging* construct refers to stakeholder involvement in implementation, such as through training and educational activities. We operationalized this construct as the Champions’ perception of their training to use the ACP video program and engagement of other facility staff in the program. *Executing* in CFIR represents how much implementation is conducted as originally planned, which we applied as ways in which Champions adhered to or customized the implementation process as originally planned. CFIR uses the construct of *reflecting and evaluating* to signify the quantitative and qualitative feedback implementers may receive (e.g., through debriefings) about the implementation process. For PROVEN, we operationalized this construct as the Champions’ perceptions of program feedback, such as that provided in the regular conference calls.

### Analysis

Three trained qualitative researchers (J.P., P.C., L.B.) conducted thematic analysis of the interview data with a hybrid deductive/inductive approach with the CFIR framework guiding the deductive analysis. Data from the two HCS and from 4-month and 15-month interviews were grouped together for analysis. Interviews were digitally-recorded and professionally transcribed. NVivo 11 software (QSR International; Melbourne, Australia) was used to manage data.

Analysis proceeded through three phases. In the first phase, J.P., P.C., and L.B. deductively developed a preliminary, structured codebook based upon CFIR domains. In a second phase, the codebook was refined inductively to ensure inclusion of constructs not within each CFIR domain. J.P. and P.C. pursued this by: 1) independently coding data by blocks of text, and 2) holding consensus meetings after coding six transcripts at a time. These coders noted both positive and negative cases of each code within the data and engaged in analytic memo writing to crystallize their thinking. The third analytic phase consisted of J.P. and L.B. iteratively reformulating inductive codes into larger domains (i.e., those formulated a priori by CFIR), paying attention to inductively derived constructs that might need to be added.

## Results

Data from 40 interviews were analyzed (4 months: HCS1: *N* = 17, HCS2: *N* = 4; 15 months: HCS1: *N* = 15, HCS2: N = 4). Champions were female (100%) and mostly social workers (87%). Table 2 presents the analytic themes that emerged in each CFIR domain as applied to PROVEN with illustrative quotes. No inductively derived constructs were necessary to supplement the CFIR domains, though not all CFIR constructs proved relevant to analysis.

**Table 2:** Analytic Themes by CFIR Construct with Illustrative Quotes

Analytic Theme	Quote
Intervention Characteristics	
Evidence Strength & Quality	
● Videos provided helpful detail and understandable framework for conceptualizing care options.	● *Champion 1*: “I just think it [the video] makes everyone more aware of what’s out there…it’s just the knowledge that there are things out there that aren’t just whether you want to be resuscitated or not…there’s a whole package of information that you really can consider.”
● Videos were valuable “openers” to ACP conversations, instigators of advance directive completion, and educational tools for future decisions.	● *Champion 2: “*And many of them, where in the past if they don’t have written healthcare directives and I give them the packet, in the past a lot of times they just kind of put it in a folder. Where now that they’re watching the video, they’re like, ‘I really need to complete these forms. You know, that video was really encouraging. I can’t wait.’” ● *Champion 3: “*I mean, everybody that’s watched it has been receptive. Like, ‘Oh, that’s good information,’ and it’s like they’re tucking it away for the future. And our long-term, the ones that we’ve done ... Like, I’m thinking of one family member in particular, it’s more they’re really kind of chewing on it for right now.”
● Videos contained a “bias” against aggressive care options.	● *Champion 3:* “We had one person [patient] say that, ‘Are they trying to tell me that I should be a DNR?’…so I would say, if I personally was gonna change anything [about the video], it would just maybe be the tone of some of the conversation.”
Relative Advantage	
● Videos’ visual nature made them particularly helpful compared to verbal conversations alone.	● *Champion 2:* “Again, it’s a great tool to have. What people most like is being able to see the visual of what CPR and what intubation is. It gives them something to visualize when they’re making their decisions. Otherwise, I think a lot of them wouldn’t always understand what we mean even though we explain it. I think seeing the video helps tremendously.”
Adaptability	
● Linguistic translations, content specific to medical condition, and both tablet and on-line access to the videos maximized the ability to adjust for stakeholder needs.	● *Champion 4: “*And for me with long term [patients], I would say actually the fact that the links are available online [works well], because most people do not want to take the time in the moment when we bring it up to view a video. They’d rather watch it from, in their own time, so having the links has been most helpful, I think for us.” ● *Champion 5:* “What has gone particularly well?...I guess I would just have to say the individualized videos that kinda coincides with what’s going on with that person at that time.”
Cost	
● Video length did not typically introduce a time burden.	● *Champion 2: “*And I do like that it’s done in a concise fashion. You know, the General Care [video] is like six and a half minutes. I like that it’s short. If it were any longer I don’t think people would watch it. The fact that it’s short, I’ve been able to entice people to watch it by telling them that it’s only a six and a half minute video. And it’s like, ‘Oh, okay. Then I’ll watch it.’”
Inner Setting	
Available Resources (within Readiness for Implementation)	
● Organizational provision of staffing and dedicated time was not necessarily sufficient for implementation efforts.	● *Champion 6:* “Time. Like I said, things have come up where I think the original champion was our DON [Director of Nursing]. Our DON left, we got a new DON. We had an acting DON, so everything was put on me. I had other things arise within our census. I couldn’t prioritize them, and like I said, I asked for help, and medical records was helping. But time would be the most challenging part.” ● *Champion 7:* “Well, it’s probably just that…not so that the videos are as long, it’s just to try to put so much in today in one little session. Sometimes if we have care plan meetings, the families use that as a bickering session, we try to inform and educate during that. Sometimes they just...I think it’s just the time.”
Networks & Communications	
● Some champions actively informed other staff of the ACP video program (e.g., through staff meetings), while others did not.	● *Champion 6:* “They know about it, the nurse and nurse practitioners. The nurses should know. Everybody knows because we’ve talked about it at full staff meetings on multiple occasions. We have the cards [with on-line links to videos]. To my knowledge, I don’t think anybody’s not aware of it unless they’re new hires.”
Compatibility (within Implementation Climate)	
● The video program could be incorporated into current ACP processes in some facilities.	● *Champion 8:* “It just becomes part of the routine. Like I said, we have a 72 h meeting and it’s part of that care plan. If we need to talk to them about advance directives, it’s just a natural kind of progression.”
Characteristics of Individuals	
Knowledge & Beliefs about the Intervention	
● Patient/family reticence to view ACP videos due to perceived lack of personal relevance or well-established advance directives was a barrier.	● *Champion 5:* “There are a few folks that, on our short term unit, you know, people who may be in their 50’s, 60’s and we see them as very ill and high risk for readmission and re-hospitalization, they do not see themselves as that, and they, some people they just say, ‘I don’t need to see that. I don’t need advance directives, I’m young, I’m not going anywhere.’” ● *Champion 9:* “The long-stay patients, it’s mostly folks that have ... their powers of attorney are activated, and they’ll [the powers of attorney] say, ‘Well, we already have this stuff. We already talked about that.’”
● Champion perceptions of the relevance of prior experience in engaging patients/families in ACP positively and negatively impacted implementation.	● *Champion 10: “*…there’s many of us that’s been in this industry for a long time, so we’ve been pretty well versed with talking to the residents at their level, and explaining advance directives, explaining hospitalization, explaining end of life care, so, not to be rude, but it’s [offering the videos] just an extra step at this point.” ● *Champion 11:* “I think sometimes it’s hard if you’re brand new in a position if you’ve never had these kinds of conversations with families - it can be hard. I know me, starting out, I had to kind of grow into being able to talk about those things, so I think the video’s really gonna help close that learning gap. And I think it’s good because it’s something that not only social workers and nurses could use, but you can just give the links to family members and they can start having that conversation.”
Individual Stage of Change	
● Champions felt that patients/family members were not always emotionally ready to engage in an ACP conversation.	● *Champion 12:* “Very much half - 50% of them are not interested. Or they don’t want to talk about it. Or they’re not ready to talk about it. So it’s a very hard conversation sometimes that we have to back off when that happen?”
Outer Setting	
External Policy & Incentives	
● External mandates of the prescribed program protocol hindered implementation efforts.	● *Champion 13: “*Um, I wish it was offered on an as-needed basis, for families who are thinking about making a change and experiencing challenges with what to do…I wish that it wasn’t something that we had to mandate to do on every new patient, and every readmission. That would definitely make it more, in my opinion, more worthwhile, to be on an as-needed basis.” ● *Champion 13: “*…it’s [ACP] something that I already cover, so it’s just more work for me to do by completing the assessment [i.e., adherence documentation]. It’s something that I address on admission, and we address it at our care plan meetings quarterly. We address it when there are changes in conditions. So it’s just more work for me.” ● *Champion 14:* “Families are gonna be up in arms! You already showed it once.”
Process	
Engaging	
● Formal Champion training, when received, was mostly perceived as effective.	● *Champion 15:* “Oh, I felt very prepared, because I watched every video so I would know what it was and the printed material was, was excellent going through that.”
● When formal training was not received, some champions and/or supervisors instituted their own informal training.	● *Champion 16:* “It was dropped on my desk with instructions! No one—I think there might have been a[n] online thing. There might have been. I don’t know. But myself and the nurse practitioner figured it out.”
● Non-Champion staff were at times tangentially involved in implementation, most often by referring patients/families to Champions when an ACP need was perceived.	● *Champion 17:* “…if they [senior leadership] want me to show the video to a patient, they will let me know. And sometimes if they have time, I’ll give it to them, but a lot of time they’ll ask me to just go ahead and do it like, when we have patients with change of conditions.”
Executing	
● Latitude to customize the implementation protocol (e.g., the way in which patients/family members were approached) maximized program outreach.	● *Champion 18*: “…[when] it’s time for them [long-stay patients] to watch them [the videos] again or if there’s a change [in status]...I might get them popcorn, Little Debbies, that kind of thing and let them sit and make an activity out of it. That’s what I do, that’s how I use it.”
Reflecting & Evaluating	
● Ongoing cross-facility conference calls were perceived as “the best part” of implementation training given the opportunity to learn from other Champions’ experiences.	● *Champion 19:* “Just getting the feedback from the other social workers or the other champions, just to make sure I was doin’ it how it should have been done, and their questions kinda helped me for the future, stuff like that. So I think the feedback was the best part of the training, ‘cause it’s, like I said, the videos are pretty self-explanatory as far as showing ‘em, but the challenges you run into, it was good to hear other ones- other centers had the same challenges, and what they were doing to kinda overcome those things.”

*ACP* advance care planning

### Domain 1: Intervention Characteristics

Intervention Characteristics were generally perceived as facilitators to implementation. Related to *evidence strength & quality,* Champions mentioned video quality, effectiveness, and validity. Many Champions emphasized the high quality of the video content. They felt the videos presented ACP information with expansive detail, helpful descriptors, and a useful framework for decision-making. The Champions stated that the video helped patients/family members, as well as themselves, better conceptualize and comprehend goals of care options. Champions described the video as an effective “tool” and “opener” to begin ACP discussions, as well as a motivating factor for patients or proxies to complete or change their advance directives. Champions also felt the videos functioned effectively as a vehicle for education and self-reflection, enabling patients’/family members’ future ACP decision-making. As for the validity of the video’s content, a contrasting case surfaced with one Champion expressing concern that the videos presented information in a biased fashion that favored less aggressive care.

Within *relative advantage,* several Champions mentioned that the visual nature of the videos was superior to verbal descriptions alone in facilitating a deeper understanding of goals of care options and informing ACP. Champions also described a number of features related to the intervention’s *adaptability* to local needs that promoted successful implementation, such as the availability of the videos in several languages. Having choice for mode of video viewing (i.e., on the tablet at the NH or on-line at a later time) was also viewed positively. Champions also liked that the ACP program offered a choice of a suite of videos, enabling them to tailor its delivery to each patient’s medical situation.

Relevant to the *cost* construct, mixed perceptions surfaced regarding how much the video(s) consumed stakeholder resources. Some Champions viewed videos’ short duration as a facilitator of implementation while a couple of others believed the videos were too long, for example, to sustain the viewer’s attention.

### Domain 2: Inner Setting

Champions depicted Inner Setting constructs (i.e., facility characteristics) as mostly barriers to implementation. A notable barrier cited by Champions was insufficiency in facilities’ *available resources*, particularly the lack of adequate time and staffing to implement the ACP video program. The finding of insufficient time for implementation remained constant even amongst those Champions who felt the videos were brief. Related to *networks & communications*, Champions had mixed perceptions. Some Champions capitalized on opportunities to communicate with other staff about the ACP video program, such as at staff meetings. On the other hand, a few Champions noted that newly hired staff remained unaware of the program due to persistent communication gaps amongst staff within the organization. In terms of *compatibility,* Champions were conflicted as to how easily the ACP video program could be integrated into established workflow. A number of Champions felt it could be easily tagged on to current facility ACP processes, while others felt that integration into existing work systems was difficult.

### Domain 3: Characteristics of Individuals

Within Characteristics of Individuals, *knowledge and beliefs about the intervention* had both facilitating and inhibiting effects upon implementation, while the *individual stage of change* was viewed primarily as a barrier. As for the first construct, Champions commonly perceived the patient’s and/or family member’s refusal to view a video as stemming from his/her belief that ACP lacked personal relevance, both among relatively younger or short-stay patients with less advanced disease, as well as long-stay patients who already had well-established advance directives. Also related to *knowledge and beliefs*, Champions who believed they were already skilled in the ACP process thought the video program created extra, unnecessary work. Alternatively, a few Champions stated that the videos could serve as a valuable learning tool for inexperienced providers. Within the *individual stage of change* construct, Champions portrayed patients/family members as not always emotionally ready to engage in an ACP discussion, impeding Champions’ ability to maximize patient/family participation.

### Domain 4: Outer Setting

*External policy and incentives,* interpreted as features of the prescribed elements (i.e., mandates) of program implementation, emerged as barriers in three main ways. First, the program required Champions to offer videos to all new admissions and long-stay patients/family members. Several Champions felt implementation would have had a more meaningful impact if Champions had the option of only offering the video to those perceived as needing it. Second, Champions found that the implementation protocol had unproductive redundancies in that they were expected to offer a video to all long-stay patients every six months even if the patients’ clinical status was unchanged which they felt sometimes fostered resistance. Finally, the mandated EMR report that Champions were required to complete about whether or not they offered a video to a patient was in some instances deemed an unnecessary burden that lacked meaningful clinical purpose.

### Domain 5: Process

Champions had mixed perceptions about how the *engaging* construct influenced implementation. For the most part, engagement through formal training was viewed as straightforward, well-designed, and effective in preparing Champions. Not all Champions (e.g., newly appointed ones after a prior Champion left the facility) received formal training despite the HCS leadership’s ongoing efforts to address this gap. In such situations, informal training by the Champions themselves or their supervisors was sometimes initiated to ensure preparedness. Engaging non-Champion staff directly in the implementation process though present was infrequent. On occasion, however, non-Champion staff perceived a need for ACP for a patient/family member and then referred the individual to Champions for video administration.

Within the construct of *executing*, Champions mostly considered latitude to customize (vs. strictly adhering to) the delivery of the intervention as a facilitator. Examples of facility-initiated customization included sending families individual letters or newsletters with on-line video links, planning a “family night” of video viewing, or offering snacks to patients as they viewed the video as a group. As for *reflecting & evaluating*, a couple of Champions reported that ongoing cross-facility conference calls were “the best part” of implementation training given the opportunity to learn from other Champions’ experiences.

## Discussion

This study explored implementation of an ACP video education program in the NH setting under the rubric of a PCT from the perspective of the NH Champions charged with implementing it. Qualitative analysis, as guided by the five CFIR domains, revealed a number of facilitators and barriers. While Intervention Characteristics (e.g., adaptability of presentation mode and minimal time burden) were perceived as largely facilitative, Inner Setting (e.g., limited time available for implementation), Characteristics of Individuals (e.g., perceived patient/family readiness to engage in ACP), and Outer Setting (e.g., mandated program requirements) made implementation more challenging. The Process (e.g., the fact that the intervention could be customized) mostly strengthened implementation. Based upon this study, some of the implementation barriers identified can be resolved while others are inherent to both ACP efforts as well as pragmatic trials within the NH setting, raising questions about the feasibility of the program as currently designed.

Our findings extend the literature on ACP interventions in NHs, particularly within the context of a PCT. PROVEN’s adaptable and minimally time-intensive intervention facilitated its implementation. Indeed, research posits that simpler ACP tools (e.g., those not needing highly specialized intermediaries) will optimize implementation and stakeholder uptake and thus intervention effectiveness [31]. In contrast, implementation of multi-component interventions in NHs, of which ACP is a part, such as the INTERACT and the COSMOS programs, can be hindered by technical problems, time demands, and magnitude of needed organizational change [25, 26]. While some researchers have argued for the superior effectiveness of more complex interventions in realizing preference-aligned treatment [6], PROVEN’s future trial outcomes may further elucidate how well an individualized yet streamlined intervention achieves desired outcomes (e.g., reduced hospitalizations), especially in face of NHs’ innate contextual barriers.

A salient Inner Setting constraint in PROVEN and comparable studies relates to such contextual barriers, i.e., limited resources (inadequate staffing, significant turnover, competing work demands, and intensive time pressure) [7, 25, 26, 31–34]. In PROVEN, perceived lack of sufficient implementation time coincided even with perceptions of the intervention as brief. Indeed, while some Champions described PROVEN’s videos as compatible with usual clinical workflows, a critical element for ACP intervention effectiveness in fast-paced health care settings [31], others described easy workflow integration as hindered by time constraints. The intransigent nature of resource barriers in NHs will remain a challenge for future PCTs in this setting. This challenge may be best addressed by designing interventions that are as parsimonious as feasible [31] and engaging NH administrators and direct care staff in developing implementation strategy in its earliest stages [26, 35]. Designating multiple collaborating Champions who could represent different disciplines may be another solution.

Like PROVEN, implementation of other NH ACP interventions has been affected by stakeholder characteristics. As in PROVEN, other studies [7, 33] describe patients’ and families’ resistance to ACP discussions as due to beliefs of their personal irrelevance or insufficient emotional readiness to broach such discussions. Unlike PROVEN which relied on Champion report, these other studies found resistance related to patient/family-reported implicit values such as discomfort with a decision-making role, preference for less formalized ACP conversations, and family feelings of guilt [33]. Champions reinforced nuance, however, about reasons they perceived that patients and families might view ACP as personally irrelevant (i.e., short-stay patients finding it premature for their health status and long-stay patients/families feeling it had been previously addressed). Engaging NH patients and families as stakeholders early in future PCT designs for ACP interventions would help address these issues [35]. For example, such stakeholders could provide an insider perspective on the optimal timing, frequency, and context for intervention delivery which could enhance end-user buy-in.

In terms of the impact of Champions’ knowledge about ACP on implementation, prior work suggests that when NH staff lack knowledge and skill, their ability to conduct ACP is hindered [7, 33]. However, in PROVEN, some Champions who perceived that they were experienced in conducting ACP did not view the intervention as helpful and believed it may be more valuable to inexperienced staff. Better preparation of Champions about the rationale and intended role of PROVEN’s video program may have averted this issue, i.e., reinforcing the intention of the video was to standardize information about goals of care options and to enhance but not replace ACP conversations between providers and patients/families.

External mandates imposed by PROVEN’s implementation protocol, while perhaps originally devised to maximize uptake of the intervention, encumbered implementation. Champions perceived universal rather than “as-needed” administration a barrier, a finding echoed in studies noting the need to appropriately time ACP delivery given NH patients’ unpredictable illness trajectories [31, 36]. Protocol redundancies also generated concern among Champions in PROVEN as well as other studies [36], most notably that repeatedly approaching patients and family regarding ACP could impair ongoing end-user engagement. Future research will need to establish whether NH-based ACP intervention delivery is more effective when determined by standardized administration or by clinical discretion. In terms of adherence documentation, researchers have argued this element is key to implementing NH ACP interventions [35]; this theory has yet to be established in practice, however. In fact, both PROVEN Champions and authors of a community-based ACP RCT suggest that resource use for fidelity monitoring may compromise real-world application [37]. Indeed, PROVEN critically highlights how external mandates of the research context may constrain a PCT.

These potential constraints of the research context raise a larger question: whether protocols in real-world clinical settings would act similarly. A research protocol in a pragmatic trial (where implementation is rolled out by the HCS itself) is equivalent to policies and procedures used in real-world clinical program roll-out. These policies and procedures may also dictate standardized implementation so that an effective intervention is administered in the fashion that made it initially effective. Adherence documentation may also be required to establish accountability. As with a pragmatic trial, the implementation challenge would be to balance standardization and accountability with feasibility.

PROVEN Champions found the customizability of the implementation process (e.g., protocol adjustments at the local level to maximize patient and family outreach), enabled by the PCT design, to be a facilitator. In contrast, the process evaluation of the COSMOS PCT describes routine and systematic processes (e.g., a delineated process for family contact) as facilitators of a multicomponent, NH-based ACP intervention [25]. Further research will need to investigate these seemingly opposing findings. For now, the PROVEN experience suggests that customizing implementation processes as broadly as possible may be the most mutable target for optimizing implementation in NHs. Doing so may maximize PCT generalizability; however, future PCTs will also need to avert implementation error by ensuring that customization does not compromise essential elements of protocol fidelity.

This study has a number of limitations. We gathered minimal information on Champion demographic information, work experience, and personal beliefs and values which may have influenced their viewpoints. Our interview participants included program Champions but not patients and families themselves; thus, Champion report of patient and family perceptions of the program were indirect and perhaps not authentic. As for their own self-report, Champions may have responded to PROVEN interviewers with socially desirable responses. Facilitators and barriers associated with important macro-level concerns of policy and regulation did not emerge from our qualitative data, perhaps due to a lack of associated interview guide questions. Generalizability of this study is limited; we cannot claim to know whether our findings are transferable to PCTs within non-profit NHs nor to other long-term care settings.

## Conclusions

A promising ACP intervention may be hindered by immutable issues such as NHs’ limited resources (e.g. staff time) as well as end-user (i.e., Champion, patient, family member) characteristics. A key implication is that end-users, not just corporate-level stakeholders, should be invited to weigh in on the early phases of PCT design to guide optimal parameters (timing, frequency, context) for intervention delivery. Additionally, while this trial’s pragmatic design allowed mutable study processes which facilitated implementation, tension surfaced between the demands of empirical rigor and real-world exigencies. Accordingly, one must remain cognizant of how research demands may constrict the real-world nature of a PCT and, inversely, how implementation error may lead to false conclusions about intervention effectiveness.

## Additional files

Additional file 1: PROVEN ACP Champion Initial Interview, a file with the 4-month interview guide questions. (DOCX 33 kb)

Additional file 2: PROVEN ACP Champion Follow-up Interview #2, a file with the 15-month interview guide questions. (DOCX 32 kb)

## Data Availability

The data that support the findings of this study are available from the corresponding author [J.A.P.] upon reasonable request.

## Abbreviations

ACP: Advance care planning
CFIR: Consolidated Framework of Implementation Research
EMR: Electronic medical record
HCS: Health care system
NH: Nursing home
PCT: Pragmatic clinical trial
PROVEN: PRagmatic trial Of Video Education in Nursing Homes
RCT: Randomized clinical trial
