# Supplementary material for: Barriers and facilitators to implementing a pragmatic trial to improve advance care planning in the nursing home setting
Source: BMC Health Serv Res. 2019 Jul 29;19:527. doi: 10.1186/s12913-019-4309-5 (PMC6664774; doi:10.1186/s12913-019-4309-5)
Supplement: Supplementary file 1 — PROVEN ACP Champion Initial Interview, a file with the 4-month interview guide questions. (DOCX 33 kb) [file 12913_2019_4309_MOESM1_ESM.docx]

***A. ACP DETAILS***

1. What is **your current role** in the ACP Video Program at your center?
2. To which kinds of patients do **you personally** offer the videos?

- Admissions only
- Long-stay patients only
- BOTH admissions and long-stay patients 🡪 *Skip to Section C*
- Other: ______________________________________________________

1. In addition to interviewing you, we would like to interview the person at your center who is **primarily** **responsible** for offering videos with <**admissions** / **long-stay** patients>. Can you tell me who that is?
   1. First name: ___________________________________________________
   2. Last name: ____________________________________________________
   3. Title/role: _____________________________________________________
2. ***ACP PRACTICES BEFORE THE VIDEO PROGRAM***

*I’m going to move onto questions about your center’s approach to advance care planning* ***before*** *the ACP Video Program was introduced.*

1. Prior to the introduction of the ACP Video Program, how were ACP conversations for **new admissions** conducted at your center?

*If not volunteered, prompt:*

- Who initiated these conversations?
- When did these conversations happen?
- How were conversations documented?
- When / where were advance directives, such as do-not-resuscitate orders (known as DNRs) or do-not hospitalize orders (known as DNHs), documented?
- Are standard advance directive forms, such as **MOLST** or **POLST** forms, used?

*If needed:*

MOLST stands for Medical Orders for Life-Sustaining Treatment.

POLST stands for Physicians Order for Life-Sustaining Treatment.

1. Again, prior to the introduction of the ACP Video Program, how were ACP conversations for **long-stay** patients conducted at your center?

*If not volunteered, prompt:*

- Who initiated these conversations?
- When did these conversations happen?
- How were conversations documented?
- When / where were advance directives, such as do-not-resuscitate orders (known as DNRs) or do-not hospitalize orders (known as DNHs), documented?
- Are standard advance directive forms, such as **MOLST** or **POLST** forms, used?

*If needed:*

MOLST stands for Medical Orders for Life-Sustaining Treatment.

POLST stands for Physicians Order for Life-Sustaining Treatment.

1. Again, prior to the introduction of the ACP Video Program, did your center use the **INTERACT** program?

*If needed:*

*INTERACT is an acronym for "****Inter****ventions to* ***R****educe* ***A****cute* ***C****are* ***T****ransfers". The intervention is a quality improvement program designed to improve the identification, evaluation, and communication about changes in resident status*.

*If not volunteered, prompt:*

- Did your center use the advance care planning forms in INTERACT?

*The next question is about any* ***education or training*** *on advance care planning that existed at your center before the ACP Video Program began. We don’t mean training that you might have received for the ACP Video Program itself. Instead, we want to know what was offered, if anything, at your center before* *the ACP Video Program.*

1. Prior to the introduction of the ACP Video Program, what **education or training** did you receive on having advance care planning conversations with patients and families?

*If not volunteered, prompt:*

- Was this <education/training> required for you?
- When and how frequently did you receive the <education/training>?

***C. ACP VIDEO PROGRAM TRAINING***

*The next questions are about how* ***you*** *learned to use the ACP Video Program.*

1. What **training** did you receive in using the ACP Video Program?

*If not volunteered, prompt:*

- Did you attend in-person seminars? Online webinars? Both?
- What other forms of training were provided?
- Who provided the training?
- Were other staff members also trained? If so, who?

1. How **prepared** did you feel with using the ACP Video Program when it began?
2. What do you think were the **most valuable** parts of the training experience?
3. What do you think were the **least valuable** parts of the training experience?

*If not volunteered, prompt:*

- Do you have suggestions for **improving** the training?

1. ***IMPLEMENTING THE ACP VIDEO PROGRAM***

*Okay, the next questions are about how the ACP Video Program is going at your center.*

1. What has gone **particularly well** with using the ACP videos?

1. What have been the **biggest challenges** with using the ACP videos?
2. To the best of your knowledge, how has the experience of using the videos **differed** between **admissions** and **long-stay** patients?
3. Aside from the ACP Champions at your center, **how aware** of the ACP Video Program are **other staff** (like nurses, nurse practitioners, physicians, the medical director, or other providers)?

*If no one else is aware of it, skip to E6.*

1. Can you describe the involvement of other providers, such as nurses, nurse practitioners, physicians, the medical director, or other providers, **in** the ACP Video program?

*If not volunteered, prompt:*

- What has been their reaction to the program?

1. In general, how have **patients** at your center responded to the ACP Video Program?
2. In general, how have **patients’ families** responded to the ACP Video Program?
3. How has the ACP Video Program changed **your own experience** of having advance care planning conversations with patients and their families?
4. Do you have any questions, or is there anything else that you would like to share about the ACP Video Program?
