# Supplementary material for: Barriers and facilitators to implementing a pragmatic trial to improve advance care planning in the nursing home setting
Source: BMC Health Serv Res. 2019 Jul 29;19:527. doi: 10.1186/s12913-019-4309-5 (PMC6664774; doi:10.1186/s12913-019-4309-5)
Supplement: Supplementary file 2 — PROVEN ACP Champion Follow-up Interview #2, a file with the 15-month interview guide questions. (DOCX 32 kb) [file 12913_2019_4309_MOESM2_ESM.docx]

1. ***ACP DETAILS***
2. What is **your current role** in the ACP Video Program at your center?
3. To which kinds of patients do **you personally** offer the videos?

- Admissions only
- Long-stay patients only
- BOTH admissions and long-stay patients 🡪 *Skip to Section C*
- Other: ______________________________________________________

1. In addition to interviewing you, we would like to interview the person at your center who is **primarily** **responsible** for offering videos with <**admissions** / **long-stay** patients>. Can you tell me who that is?
   1. First name: ___________________________________________________
   2. Last name: ____________________________________________________
   3. Title/role: _____________________________________________________
2. Have you been you responsible for offering videos since the ACP Video Program **first began** at this center in the **<spring/summer> of 2016**, or did you become responsible at a **later** time?

- Responsible since it began 🡪 *Skip to Section D*
- Became responsible later 🡪 *If not volunteered, prompt:* Can you tell me about that?

***B. ACP VIDEO PROGRAM TRAINING***

*The next questions are about how* ***you*** *learned to use the ACP Video Program.*

1. What **training** did you receive in using the ACP Video Program?

*If not volunteered, prompt:*

- Did you attend in-person seminars? Online webinars? Both?
- What other forms of training were provided?
- Who provided the training?
- Were other staff members also trained? If so, who?

1. How **prepared** did you feel with using the ACP Video Program when it began?
2. What do you think were the **most valuable** parts of the training experience?
3. What do you think were the **least valuable** parts of the training experience?

*If not volunteered, prompt:*

- Do you have suggestions for **improving** the training?

***C.*** ***ACP PRACTICES OUTSIDE OF THE VIDEO PROGRAM***

*I’m going to move on to questions about your center’s approach to general advance care planning.*

1. In the last 6 months, **aside from the ACP Video Program,** have there been any **center-wide changes** in how advance care planning **conservations** are done in your center? *If yes:* Can you tell me about those changes?
2. In the last 6 months, **aside from the ACP Video Program,** have there been any **center-wide changes** in how advance directives, such as do-not-resuscitate orders (known as DNRs) or do-not hospitalize orders (known as DNHs), are typically **documented** for a patient?
   *If yes:* Can you tell me about those changes?

***D. IMPLEMENTING THE ACP VIDEO PROGRAM***

*Okay, the next questions are about how the ACP Video Program has been going at your center.*

1. What has gone **particularly well** with using the ACP videos?

1. What have been the **biggest challenges** with using the ACP videos?
2. To the best of your knowledge, how has the experience of using the videos **differed** between **admissions** and **long-stay** patients?
3. In your role as champion, what actions do you to take to encourage or support the use of the ACP Video Program by staff in your center?

*If not volunteered, prompt:*

a) How do you reinforce previous formal training efforts?

b) How do you promote staff buy-in?

c) How do you monitor program success?

d) How do you provide feedback to staff on program success?

1. Aside from the ACP Champions at your center, **how aware** of the ACP Video Program are **other staff** (like nurses, nurse practitioners, physicians, the medical director, or other providers)?

*If no one else is aware of it, skip to E6.*

1. Can you describe the involvement of other providers, such as nurses, nurse practitioners, physicians, the medical director, or other providers, **in** the ACP Video program?

*If not volunteered, prompt:*

- What has been their reaction to the program?

1. In general, how have **patients** at your center responded to the ACP Video Program?
2. In general, how have **patients’ families** responded to the ACP Video Program?
3. How has the ACP Video Program changed **your own experience** of having advance care planning conversations with patients and their families?

***E. IMPRESSION OF THE ACP VIDEO PROGRAM***

The next questions are about **your overall impression** of the ACP Video Program.

1. How do you think the ACP Video Program has **changed** advance care planning at your center?
2. With time, has it become **easier or more difficult** to integrate the ACP Video Program into your center? What’s **happening** at your center that gives you that impression?
3. Would you **recommend** the ACP Video Program to other skilled nursing facilities? For what **reasons** do you feel that way?

1. How could the ACP Video Program be **improved** for ongoing use **in the future**?

*We are approaching the final section of our interview.*

1. Describe your experience using National Healthcare Decisions Week in April 2017 to introduce the ACP videos to patients and families. What was helpful about having a special week devoted to health care decisions in introducing the videos? What was not helpful?
2. Around April 2017 you were provided with a list of long-stay residents at your facility who have not yet seen a video. Talk about how this list was used at your facility. What was helpful about getting the list? What was not helpful?
3. Do you have any questions, or is there anything else that you would like to share about the ACP Video Program?
